# Editorial: Novel methods in embodied and enactive AI and cognition

**DOI:** 10.3389/fnbot.2023.1162568

**Published:** 2023-03-07

**Authors:** Fabio Bonsignorio

**Affiliations:** ^1^AIFORS Lab, FER, University of Zagreb, Zagreb, Croatia; ^2^Heron Robots, Genoa, Italy

**Keywords:** novel paradigms in embodied AI, novel paradigms in enactive AI, embodiment, self-organization, symbol anchoring, information driven self organization, Deep Learning, frame of reference issue

## 1. Introduction

Many people still identify AI with GOFAI (Good Old Fashioned Artificial Intelligence), broadly speaking “functionalist” and “symbolic” AI, or Machine Learning or Deep Learning (DL) (Hinton and Salakhutdinov, 2006), mainly due to recent successes in object and speech recognition and other tasks. DL methods, and recently transformers and generative AI, text-to-image generation tools (such as MidJourney, StableDiffusion, and Dall-E), and Large Language Models (LLMs) (such as ChatGPT) have proven to be very powerful in a wide range of applications. However, they usually need to be trained on vast datasets (hence the phrase “Big Data”); they work less well and with lower effectiveness when available training data are less abundant, or when intelligent behaviors of physical agents in a physical environment require real-time reactions to a sensorimotor flow affected by agent behavior. When humans are involved, the “explainability” of AI reasoning is of great importance and is often weak. This is in part due to the fact that Deep Learning has been initially applied for the indexing of large data sets of images and sound samples, and for profiling of online customers of marketplace platforms and users of social networks. There might be deeper reasons for that. In nature, cognition and intelligence follow a completely different paradigm than in current mainstream AI and robotics. They are embedded in a physical system (a body) (Pfeifer and Bongard, 2006), emerging bottom-up from the interaction of large numbers of loosely coupled components (Bonsignorio, 2007), and they are usually associated with life. In contrast, the “mechatronics + machine learning” paradigm, which is still used to build mainstream robots, implements top-down controls and keeps the body well divided from the mind (following the views of seventeenth-century European philosopher Descartes). Frame of Reference (F-O-R) issues in AI (Dennett, 1987) also affect Deep Learning models; human “tagging” in model training or in LLM tuning *via* Reinforcement Learning by Human Feedback (RLHF) are clear examples of this. Several different approaches have been tried in order to frame the problem of designing physical agents acting in the real world in a way directly inspired by natural intelligence. Many researchers have seen in soft robotics a way to pursue approaches more closely related to what we observe in nature without questioning the widely accepted paradigms. In many cases, the models of robots make precise assumptions about the body and the environment, such as that the body is made up of a known number of rigid objects connected to one another *via* joints of a known type, like those in the skeleton (Bongard et al., 2006; Hersch et al., 2009; Sturm et al., 2009; Koos and Mouret, 2011). Some bolder research has employed other approaches, including self-organizing maps or Kohonen-type unsupervised machine learning (Asada et al., 2009; Mori and Kuniyoshi, 2010). Probabilistic measures like Crutchfield information metrics have been used to determine topological relationships between sensors and actuators (Olsson et al., 2004; Kaplan and Hafner, 2005; Schatz and Oudeyer, 2009). New measures (Zenil et al., 2013) have been developed to help detect life (whether artificial or natural); see Terrazas et al. (2013). Kohonen's Self-Organizing Maps have been used to learn visuomotor coordination (Ritter et al., 1989). Kuiper's Spatial Semantic Hierarchy shows that intrinsic knowledge alone may be used to reconstruct an instructive and demanding sensorimotor contingency. Pierces and Kuipers concepts have been extended with even weaker assumptions through analysis of sensorimotor variable distances using statistically based information-theoretic metrics (Crutchfield, 1990; Olsson et al., 2004). Tanev et al. (2005) and Prokopenko (2013, 2014) propose to exploit information-driven self-organization. In this context, Bonsignorio (2013) recommends leveraging Lie Groups to reduce the computational load of information-driven self-organization, building on work by Chirikjian and Burdick (1994) and Chirikjian (2010). One of main missions of the Human Brain Project, a flagship EU project, is dedicated to neurorobotics, a new field aiming to merge neurosciences, AI, and robotics. Emergentist approaches to embodied/enactive AI are in principle capable of escaping the limitations of “symbolic” and “functionalist” AI and ML/DL (above all, the F-O-R issue). However, the problem of anchoring “pseudo?-symbolic” concise “pseudo?-representations” to the sensorimotor flow has still to be solved. The line between what is “pre-programmed” and “learned” in natural intelligent agents and where this should be in artificial ones has yet to be fully grasped; see Cangelosi and Asada (2022). Promising approaches leveraging system dynamics have been proposed, recently by Billard et al. (2022).

## 2. Contributed articles

The contributed papers cover some of the more challenging open questions in the area of Embodied and Enactive AI and propose some original approaches. Scarinzi and Cañamero argue that “artificial emotions” are a necessary tool for an agent interacting with the environment. Hernandez-Ochoa point out the potential importance and usefulness of the evo-devo approach for artificial emotional systems. The problem of anchoring a symbolic description to a neural encoding is discussed by Katz et al., who propose a “neurocomputational controller” for robotic manipulation based on a “neural virtual machine” (NVM). The NVM encodes the knowledge of a symbolic stacking system, but can then be further improved and fine-tuned by a Reinforcement Learning procedure. This is an approach attempting to bridge “symbolic descriptions” with data-driven approaches. In Hinrichs et al., the authors show via a thorough data analysis how “meaning,” as it is understood by us humans in natural language, is actually an unstable ground for symbolic representations, as it shifts from language to language. An early stage controller inspired by Piaget's schemas is proposed by Lagriffoul. The relevance of Piaget's work, which provides an insightful analysis of cognitive development in human children, has been recognized for example in Bonsignorio (2007).

We should not underestimate the importance of establishing suitable, cheap, and easily customizable testing platforms for real-world testing of scientific ideas in embodied and enactive AI and cognition. This is addressed by Stoelen et al..

## 3. Discussion

Despite some successes, much work still remains to be done (Ackerman and Guizzo, 2015; Yanco et al., 2015). In particular, “tabula rasa” (“blank slate”) methods have shown serious limitations in terms of moving beyond “shallow understanding” to the deeper understanding that is required to achieve adaptive, resilient, and trustworthy behaviors in physical agents (Marcus and Davis, 2019). Deep Learning (DL) algorithms cannot infer high-level representations or causal links, or make strong anticipatory actions. Might more abstract approaches, reproposing hard (symbolic) modeling approaches from a system theory point of view, such as that of the Coresense project, be merged with “emergentist” data-driven pipelines? To overcome their current limitations, the fields of AI and robotics need to move from a “Cartesian” and “clock-like” mechatronic-plus-machine- learning paradigm to a radically new one, based on the reverse-engineering of animal intelligence and cognition. The new approach will need to be strongly interdisciplinary, as it will have to borrow principles and methods from—to name a few fields—AI, neuroscience, artificial life, and synthetic biology.

## Author contributions

The author confirms being the sole contributor of this work and has approved it for publication.
